# Gut and flesh microbiome sequencing of the Bangladesh national fish hilsa (*Tenualosa ilisha*)

**DOI:** 10.1128/MRA.00448-23

**Published:** 2023-09-25

**Authors:** Tofazzal Islam, M. Nazmul Hoque

**Affiliations:** 1 Institute of Biotechnology and Genetic Engineering (IBGE), Bangabandhu Sheikh Mujibur Rahman Agricultural University (BSMRAU), Gazipur, Bangladesh; 2 Department of Gynecology, Obstetrics and Reproductive Health, BSMRAU, Gazipur, Bangladesh; Montana State University, Bozeman, Montana, USA

**Keywords:** *Tenualosa ilisha*, Hilsa flesh, gut microbiome, metagenomics

## Abstract

The gut and flesh microbiome of the national fish of Bangladesh, *Tenualosa ilisha*, were analyzed using 16S rRNA gene sequencing. Our findings revealed a significant microbial disparity between sample categories and the habitat of hilsa fish, which will serve as a valuable foundation for further comprehensive studies on the hilsa microbiome.

## ANNOUNCEMENT

Hilsa (*Tenualosa ilisha*) is the national fish of Bangladesh. It contributes ~12% of the total fish production and 1.15% GDP of the country (1). It is widely distributed in Southeast and South Asia (2, 3) and found in coastal areas, estuaries, brackish, and freshwater rivers (3, 4). Hilsa is an anadromous marine fish that comes to freshwater in rivers during spawning. Gut microbiota, which modulate host homeostasis, immunity, and fitness (5, 6), have been analyzed in many fish species (7, 8) but rarely in the hilsa fish (1). Nothing is known about the gut and flesh microbiome of hilsa, which is known to be free from diseases. This study aimed to analyze the gut and flesh microbiome of hilsa fish using 16S rRNA gene amplicon sequencing.

Eighteen adult hilsa fishes were collected and frozen from September to October 2020 from five different sites in Bangladesh (Table 1). Sampling of their intestinal contents (*n* = 15) and flesh (*n* = 3) was performed within 48 h after the collection. Fish scales from the body of all individual fishes were removed by sterile forceps under aseptic conditions, and fleshes (100 mg) and lower stomach contents (100 mg) from each fish were collected. Genomic DNA was extracted from each specimen after grinding (flesh) and homogenization (gut content) using DNeasy Blood and Tissue Kit (Qiagen, UK) following the manufacturer’s instructions. A NanoDrop 2000c spectrophotometer was used to check the purity and concentration of DNA. The amplicon libraries were prepared using Nextera XT Index Kit (Illumina Inc.) as per the 16S amplicon sequencing library preparation protocol, and V3–V4 regions were amplified through a 40-cycle PCR using 341F and 806R primers (9). Paired-end sequencing (2 × 150 bp, 300 cycles) was performed on the Illumina MiniSeq sequencer. FastQC v0.11.9 (10) was used to check the quality of the reads, while Trimmomatic v0.39 (11) removed Illumina adapters, known Illumina artifacts, and phiX reads. The demultiplexed sequencing data were processed using QIIME 2 (2023.2.0) and associated plugins (12). Sequences showing ≥98% similarity were grouped into operational taxonomic units (OTUs) using the SILVA database v.138 (13). Default parameters were used for bioinformatic analyses, except where otherwise stated.

**TABLE 1:** Summary of metadata and SRA accession numbers of the 16S rRNA amplicon sequences of hilsa fish gut and flesh samples, along with OTUs mapped against bacterial taxa

Sample ID	Collection site	Coordinate	Habitat	Source	No. of raw reads	No. of mapped reads	No. of observed OTUs	SRA accessions
CG1	Confluence of Meghna and Padma River, Chandpur	23.2321° N, 90.6631° E	Freshwater	Gut	325,812	33,295	22	SRR24402593
CG2	Confluence of Meghna and Padma River, Chandpur	23.2321° N, 90.6631° E	Freshwater	Gut	140,124	12,418	22	SRR24402592
CG3	Confluence of Meghna and Padma River, Chandpur	23.2321° N, 90.6631° E	Freshwater	Gut	119,676	4,738	10	SRR24402608
CG4	Confluence of Meghna and Padma River, Chandpur	23.2321° N, 90.6631° E	Freshwater	Gut	697,544	291,731	23	SRR24402607
CG5	Confluence of Meghna and Padma River, Chandpur	23.2321° N, 90.6631° E	Freshwater	Gut	131,276	9,514	20	SRR24402606
RG3	Padma River, Rajshahi	24.3745° N, 88.6042° E	Freshwater	Gut	194,464	2,256	18	SRR24402605
MG1	Meghna River, Munshiganj	23.5422° N, 90.5305° E	Freshwater	Gut	165,096	1,333	15	SRR24402604
MG2	Meghna River, Munshiganj	23.5422° N, 90.5305° E	Freshwater	Gut	126,908	7,353	15	SRR24402602
MG4	Meghna River, Munshiganj	23.5422° N, 90.5305° E	Freshwater	Gut	260,420	3,077	11	SRR24402601
PG1	Payra River, Patuakhali	22.3586° N, 90.3317° E	Brackish water	Gut	149,988	611	23	SRR24402600
PG2	Payra River, Patuakhali	22.3586° N, 90.3317° E	Brackish water	Gut	119,052	1,142	11	SRR24402598
PG3	Payra River, Patuakhali	22.3586° N, 90.3317° E	Brackish water	Gut	361,312	8,111	20	SRR24402599
PG5	Payra River, Patuakhali	22.3586° N, 90.3317° E	Brackish water	Gut	178,372	3,982	18	SRR24402597
XG1	Bay of Bengal, Cox’s Bazar	21.4272° N, 92.0058° E	Marine water	Gut	112,684	509	16	SRR24402596
XG3	Bay of Bengal, Cox’s Bazar	21.4272° N, 92.0058° E	Marine water	Gut	151,480	260	14	SRR24402595
CF4	Confluence of Meghna and Padma River, Chandpur	23.2321° N, 90.6631° E	Freshwater	Flesh	180,944	4,994	16	SRR24402609
CF5	Confluence of Meghna and Padma River, Chandpur	23.2321° N, 90.6631° E	Freshwater	Flesh	106,604	2,226	14	SRR24402603
PF4	Payra River, Patuakhali	22.3586° N, 90.3317° E	Brackish water	Flesh	174,852	2,739	17	SRR24402594

The 16S rRNA amplicon sequencing of 18 samples generated 3,696,608 raw reads (average: 205,367 reads/sample), of which 390,289 quality reads (10.56%) mapped to 325 OTUs of bacteria. The rest of the reads (90%) were unassigned to any OTUs and might come from host DNA. Among these, 67 and 258 OTUs were identified in flesh and gut samples, respectively, through the SILVA database v.138 (Table 1). These OTUs were represented by six phyla, nine classes, 19 orders, 26 families, and 40 genera of bacteria. Firmicutes constituted >75.0% of the hilsa bacteriome, with 78.17% and 2.86% relative abundances in the gut and flesh, respectively. *Vagococcus* (67.02%), *Morganella* (13.25%), *Enterobacter* (5.74%), *Plesiomonas* (3.51%), *Shigella* (1.75%), *Clostridium* (1.60%), *Klebsiella* (1.02%), and *Serratia* (1.0%) were the top abundant bacterial genera detected in hilsa fish, with distinct variations in their relative abundances according to the sample categories (gut and flesh; *P*  =  0.0127; Kruskal Wallis test). The genomic data of the flesh and gut bacteriome of hilsa fish revealed in this study has laid the foundation for shedding light on the microbiome of this economically important trans-boundary fish.

## Data Availability

The 16S rRNA gene amplicon sequencing data are available at the NCBI Sequence Read Archive (SRA) under BioProject accession number PRJNA964437. The accession numbers for all SRA experiments are listed in Table 1.
